# Revealing immune responses in the *Mycobacterium avium* subsp. *paratuberculosis*-infected THP-1 cells using single cell RNA-sequencing

**DOI:** 10.1371/journal.pone.0254194

**Published:** 2021-07-02

**Authors:** Hong-Tae Park, Woo Bin Park, Suji Kim, Jong-Sung Lim, Gyoungju Nah, Han Sang Yoo

**Affiliations:** 1 Department of Infectious Disease, College of Veterinary Medicine, Seoul National University, Seoul, Korea; 2 Genome Analysis Center, National Instrumentation Center for Environmental Management, Seoul National University, Seoul, Korea; Cornell University, UNITED STATES

## Abstract

*Mycobacterium avium* subsp. *paratuberculosis* (MAP) is a causative agent of Johne’s disease, which is a chronic and debilitating disease in ruminants. MAP is also considered to be a possible cause of Crohn’s disease in humans. However, few studies have focused on the interactions between MAP and human macrophages to elucidate the pathogenesis of Crohn’s disease. We sought to determine the initial responses of human THP-1 cells against MAP infection using single-cell RNA-seq analysis. Clustering analysis showed that THP-1 cells were divided into seven different clusters in response to phorbol-12-myristate-13-acetate (PMA) treatment. The characteristics of each cluster were investigated by identifying cluster-specific marker genes. From the results, we found that classically differentiated cells express *CD14*, *CD36*, and *TLR2*, and that this cell type showed the most active responses against MAP infection. The responses included the expression of proinflammatory cytokines and chemokines such as *CCL4*, *CCL3*, *IL1B*, *IL8*, and *CCL20*. In addition, the Mreg cell type, a novel cell type differentiated from THP-1 cells, was discovered. Thus, it is suggested that different cell types arise even when the same cell line is treated under the same conditions. Overall, analyzing gene expression patterns via scRNA-seq classification allows a more detailed observation of the response to infection by each cell type.

## Introduction

*Mycobacterium avium* subsp. *paratuberculosis* (MAP) is a causative agent of Johne’s disease, which is a chronic and debilitating disease in ruminants. MAP is also considered to be a possible cause of Crohn’s disease in humans. Crohn’s disease is a chronic inflammatory bowel disease characterized by intermittent diarrhea, weight loss, bleeding, and bowel obstruction [1]. Because the clinical features are similar between Johne’s disease and Crohn’s disease, Crohn’s disease has long been hypothesized to be associated with MAP infection [2]. Despite controversy surrounding whether MAP causes Crohn’s disease, there are many positive links between Crohn’s disease and MAP infection [3–6]. Many studies have revealed that MAP infections are significantly more common in Crohn’s disease patients than in healthy individuals [3,4]. In addition, MAP-specific antibiotic treatment led to the remission of active Crohn’s disease [1,7,8]. However, few studies have focused on the interactions between MAP and the human immune system to elucidate the pathogenesis of Crohn’s disease.

Similar to other pathogenic mycobacteria, the major pathogenic mechanism of MAP infection in cattle is persistent survival in phagocytic cells, which induce granuloma formation. Infected macrophages and dendritic cells induce Th17-like immune responses by secreting IL-6 and IL-23, which are polarizing factors of IL-17-producing T helper cells [9,10]. IL-17, along with TNF-α, is a major cytokine that contributes to the development and maintenance of granuloma [11,12]. Because phagocytic cells, such as macrophages, play a pivotal role in directing the adaptive response by interacting with and processing antigens, investigating the responses of macrophages against MAP provides information about which “weapons” are used for defense against this intracellular pathogen.

THP-1 is a human leukemia monocytic cell line that can be differentiated into a macrophage-like phenotype by stimuli such as phorbol-12-myristate-13-acetate (PMA) [13]. For the effective differentiation of THP-1 cells into macrophages, various concentrations of PMA and treatment times have been tested by many researchers [14–16]. In a recent study, it was determined that THP-1 cells differentiated under specific conditions have similar characteristics to human monocyte-derived macrophages (MDMs) through the Salmonella infection test [16]. The authors suggested that extended resting time after PMA treatment induces a more macrophage-like phenotype characterized by the surface expression of CD11b and CD14. However, in previous studies, it was found that not all cells uniformly exhibited one characteristic, even under the same treatment conditions. Because of the heterogeneity of cells according to the differentiation process, we hypothesized that the response to MAP infection would also differ depending on the characteristics of the cells.

Single-cell RNA-seq (scRNA-seq) is a powerful tool for cell characterization as it enables clustering based on single-cell transcripts. Recently, the change in the immune response according to disease severity was investigated using scRNA-seq analysis of the immune cells of COVID-19 patients [17,18]. The authors compared the gene expression patterns of immune cell clusters divided by scRNA-seq analysis and suggested that disease severity is affected by the predominance of innate and adaptive immune responses. In addition, ecological analysis of pulmonary granuloma against *M*. *tuberculosis* (Mtb) infection in an animal model was analyzed based on scRNA-seq to investigate the mechanisms related to the occurrence and maturation of granuloma [19]. Through this analysis, it was confirmed that mast cells and plasma cells were expanded in the granuloma of high-burden lesions.

In the present study, the differentiation state of THP-1 cells in specific PMA treatment conditions was determined by scRNA-seq analysis. Through clustering analysis, cells expressing similar transcripts were classified into specific clusters, and differentially expressed genes (DEGs) according to MAP infection for each cluster were analyzed. Through the analysis, it was confirmed that THP-1 cells differentiated into various types or to different degrees of differentiation after PMA treatment, and the response to MAP at the initial stage of infection was also slightly different for each cell type.

## Materials and methods

### Ethics statement

This study was conducted in an approved facility in strict accordance with all university and federal regulations. All experiments were reviewed and approved by the Institutional Biosafety Committee (Approval no. SNUIBC-R200324-1).

### Cell culture and infection

THP-1 cells were cultured in RPMI 1640 supplemented with 10% heat-inactivated FBS (Gibco) and 1% penicillin/streptomycin at 37°C in humidified air with 5% CO_2_. Differentiation of THP-1 cells into macrophages was performed by stimulation with 50 ng/ml PMA (Sigma Co., St. Louis, MO, USA). After 72 h of incubation, the cells were washed twice with FBS-free RPMI 1640 medium and incubated with 5% FBS-RPMI 1640 medium without antibiotics for 24 h before the experiments. Differentiated THP-1 cells (5 × 10^6^ cells) were inoculated with MAP strain K-10 at a multiplicity of infection (MOI) of 10:1 and incubated for 3 h. Cells were then detached from the culture dish and washed with 1 × DPBS supplemented with 0.04% bovine serum albumin (BSA). Single-cell suspensions of MAP-infected and noninfected control THP-1 cells were subjected to scRNA-seq.

### Single-cell RNA-sequencing and data processing

To generate single-cell gel beads in the emulsion (GEMs), single-cell suspensions were loaded onto a Chromium Controller (10x Genomics) using Chromium Next GEM Single Cell 3ʹ GEM kit v3.1. Approximately 3,000 target cells were recovered for each group, and a scRNA-seq library was constructed using Chromium Next GEM Single Cell 3ʹ Library Kit v3.1. All preparation steps were performed according to the manufacturer’s specifications. Constructed libraries were sequenced using an Illumina NovaSeq6000 (Illumina) with 150-bp paired-end reads. Sequencing data were processed using Cell Ranger software (v4.0.0) and aligned to the human reference genome GRCh37/hg19. Downstream analysis of filtered gene expression matrices was performed using the Seurat package (v4.0.1) running in R software (v4.0.5).

In the process of analysis, mitochondria-rich cells and cells with low UMI counts indicating low-quality cells were trimmed using the following criteria: 2.5 < mitochondria (%) < 9 and 2000 < nFeatureRNA < 6000. The trimmed data was normalized by “LogNormalize” method. The normalized data from each group of cells (control vs. infected) were then integrated by following the described at “https://satijalab.org/seurat/articles/integration_introduction.html”. The integrated dataset was further processed to cluster the cells using linear dimensional reduction (“RunPCA” function with default option) and nonlinear dimensional reduction (“RunUMAP” function with following option: dims = 1:30), followed by clustering analysis (“FindNeighbors” and “FindClusters” function with following option: dims = 1:30, resolution = 0.5).

### Differential gene expression analysis

Cluster-specific biomarkers were sought using the “FindAllMarkers” function in the Seurat package (v4.0.1) with the default option. The expression level of the top 10 featured genes in each cluster was visualized by a heatmap, and several genes among the top 10 genes were selected as candidates for cluster identification markers. DEG analysis between infected and control cells specific to each cluster was further performed using the Wilcoxon test included in the “FindMarkers” function of the Seurat package. DEGs in specific cluster compared to other was also calculated with “FindMarkers” function. In this case, a second identity class for comparison was set to NULL to use all other cells for comparison.

### Trajectory analysis

The trajectory analysis was performed by following the instructions described at “https://satijalab.org/signac/articles/monocle.html”. Briefly, single-cell trajectory analysis was performed using the Monocle 3 (v0.2.3.0) package running in R software (v4.0.5). Single-cell objects processed by the Seurat package were converted to “CellDataSet” using SeuratWrappers (v0.3.0). The converted object was loaded onto Monocle 3, and the trajectory was built. Then, pseudotime estimation was performed using the “order_cells” function.

## Results

### Clustering analysis

**Fig 1:**
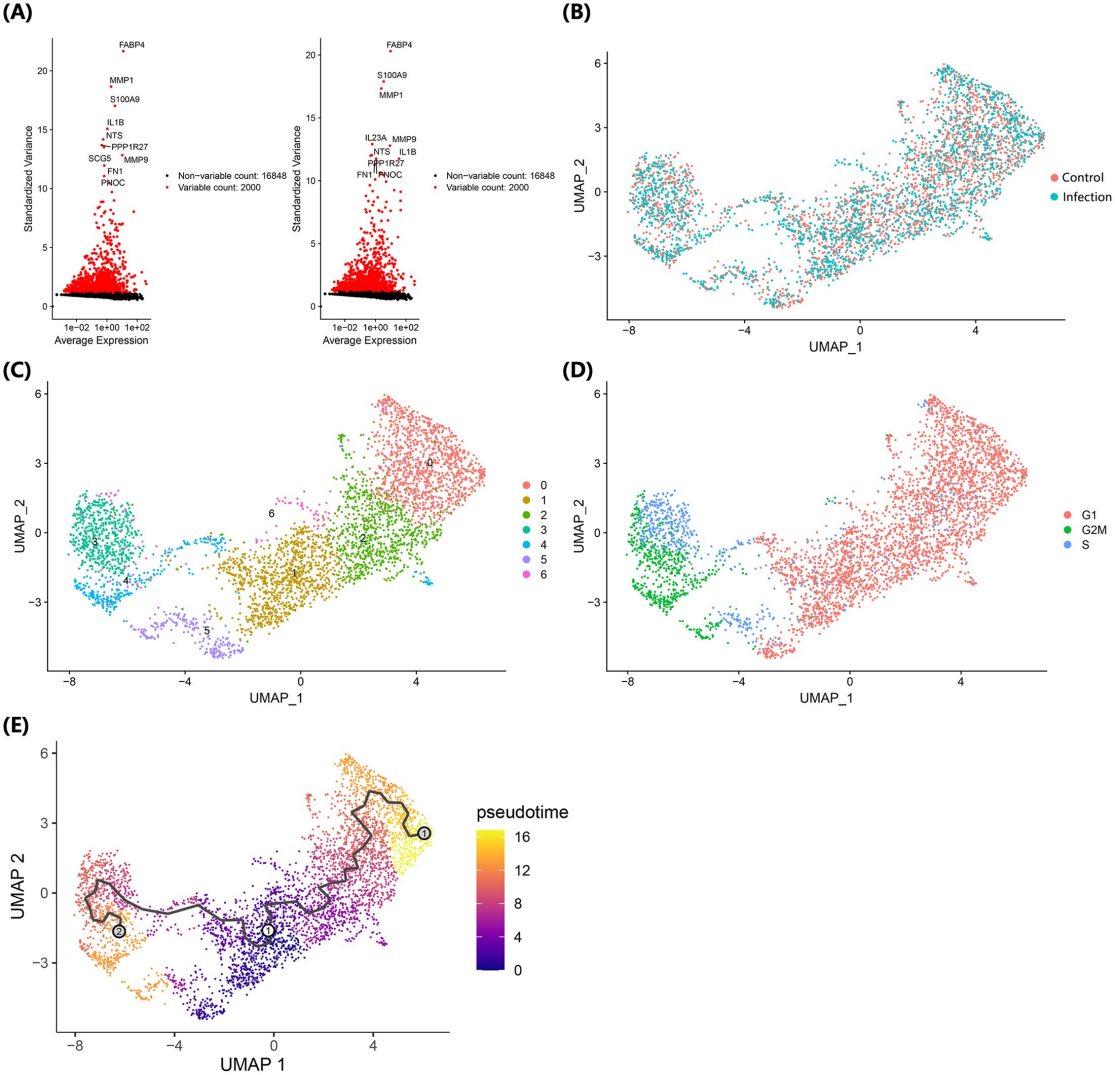
Single-cell transcriptional profiling of MAP-infected and control THP-1 cells after PMA treatment. Genes that showed variable features throughout the cell population and the top 10 features of the control group (left) and infection group (right) are described (A). Using the Seurat package, the UMAP plot of single cells was described with discrimination of the control and infection groups (B), formation of clusters (C), and cell cycle (D). Using the Monocle 3 package, the pseudotime of single cells was predicted with a trajectory analysis (E).

**Fig 2:**
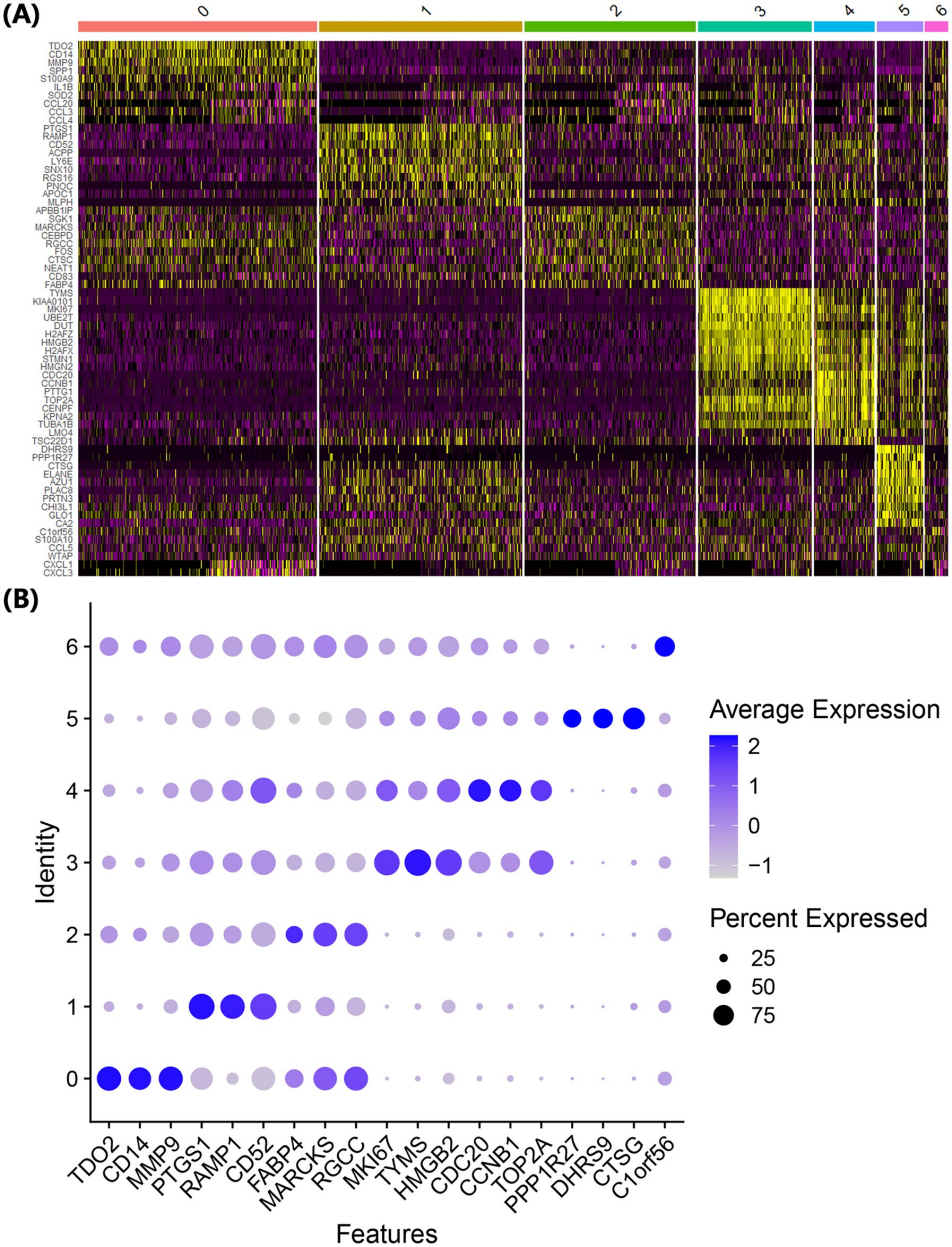
Expression of cluster-specific marker genes. Genes showing markedly different expression in each cluster are indicated by a heatmap (A), and the expression of cluster-specific marker candidate genes was compared using a dot plot (B).

scRNA-seq analysis of THP-1 cells was performed 3 h after MAP infection. From the normalized gene expression features, highly variable features were similar in both the control and infection groups, but the infection group showed specific expression of IL23A (Fig 1A). The cause of similar features in the two groups was thought to be the differentiation-related characteristics of THP-1 cells following PMA treatment, and *IL23A* expression was presumed to be a characteristic of MAP infection. After integration of the data between the two groups, a total of 7 clusters were identified as a result of clustering analysis (Fig 1B and 1C). Since several cell clusters were identified in one cell line, the distinction into different clusters was thought to represent differentiation of the cells into various types in response to the PMA treatment. According to the cell cycle-related classification data, the cells could be broadly classified into two clusters, clusters 0, 1 and 2 and clusters 3 and 4, whereas clusters 5 and 6 consisted of all cells in the G1, G2/M, and S cycles (Fig 1D). Displaying the main marker genes that classified each cluster on the heatmap divided the clusters into two groups similar to the cell cycle-related classification (Fig 2A). The results showing the expression level of the main genes identified as marker genes in this analysis by dot plot also showed a pattern similar to the previously confirmed results (Fig 2B). Cluster 5 showed a pattern of gene expression that distinguished it from the other clusters.

To clarify the cell type of each cluster, the main marker genes that were sorted by the relative gene expression (log2-fold-change) and percentage of cells that expressed the gene in each cluster were analyzed (S1 File). In cluster 0, the expression of *CD14*, which is a known differentiation marker of THP-1, was higher than that of other clusters, and the expression of genes such as *TDO2*, *MMP9*, *SPP1*, and *S100A9* were also higher than in other clusters (Fig 2A and 2B). CD14 expression increases over time in macrophages that differentiate from THP-1 cells in response to PMA treatment [16]. Therefore, cells in cluster 0 were determined to be classically differentiated macrophages. Cells in cluster 1 showed higher expression of *PTGS1*, *RAMP1*, *CD52*, and *ACPP* than the other clusters. Interestingly, when the expression levels of the marker genes of cluster 0 and cluster 1 were compared, the opposite pattern was observed (Fig 2B). In particular, *RAMP1* gene expression tended to be prominent only in cluster 1; thus, *RAMP1* is likely to have significance as a marker that differentiates cluster 1 cells from classically differentiated cells. Cluster 2 cells were presumed to have an intermediate characteristic between clusters 0 and 1 from the expression trends of the marker genes. In particular, assuming that the expression of *CD14* etc. increases with time after PMA treatment, the expression of these genes can be seen as indicative of the degree of differentiation in the order of clusters 1, 2, and 0. To confirm this, trajectory analysis was performed, and the predicted pseudotime value increased in both directions towards clusters 0 and 4 when cluster 1 was set to 0 hours (Fig 1E).

Since the genes identified in clusters 3 and 4 are mainly involved in the cell cycle, these clusters appear to consist of proliferating cells, and as shown in UMAP, the distinction between the G2/M and S phase appears to be the main distinction point. Cluster 5 cells are characterized by the expression of *DHRS9*, *PPP1R27* and *CTSG*, which are rarely expressed in other clusters, and *DHRS9* is a known stable marker of regulatory macrophages [20]; thus, cluster 5 was considered to be a group of Mreg cells. Finally, in cluster 6 cells, only *C1orf56* was significantly expressed in the infection group (log_2_FC > 1.0), and no genes specifically expressed in the cluster were found.

### Differential gene expression analysis with a single-cell transcriptome

Profiling analysis was performed on genes differentially expressed in the infection group compared to the control for each cluster (S2 File). Interestingly, although THP-1 cells were classified into different clusters according to various gene expression characteristics following PMA treatment, the main responses of the cells to MAP infection were similar. For example, DEG analysis of infected vs. control cells for each cluster confirmed that *IL8* gene expression was significantly increased in all clusters (adjusted p-value < 0.05). However, there were significant differences in the expression levels of the main genes associated with each cluster.

**Fig 3:**
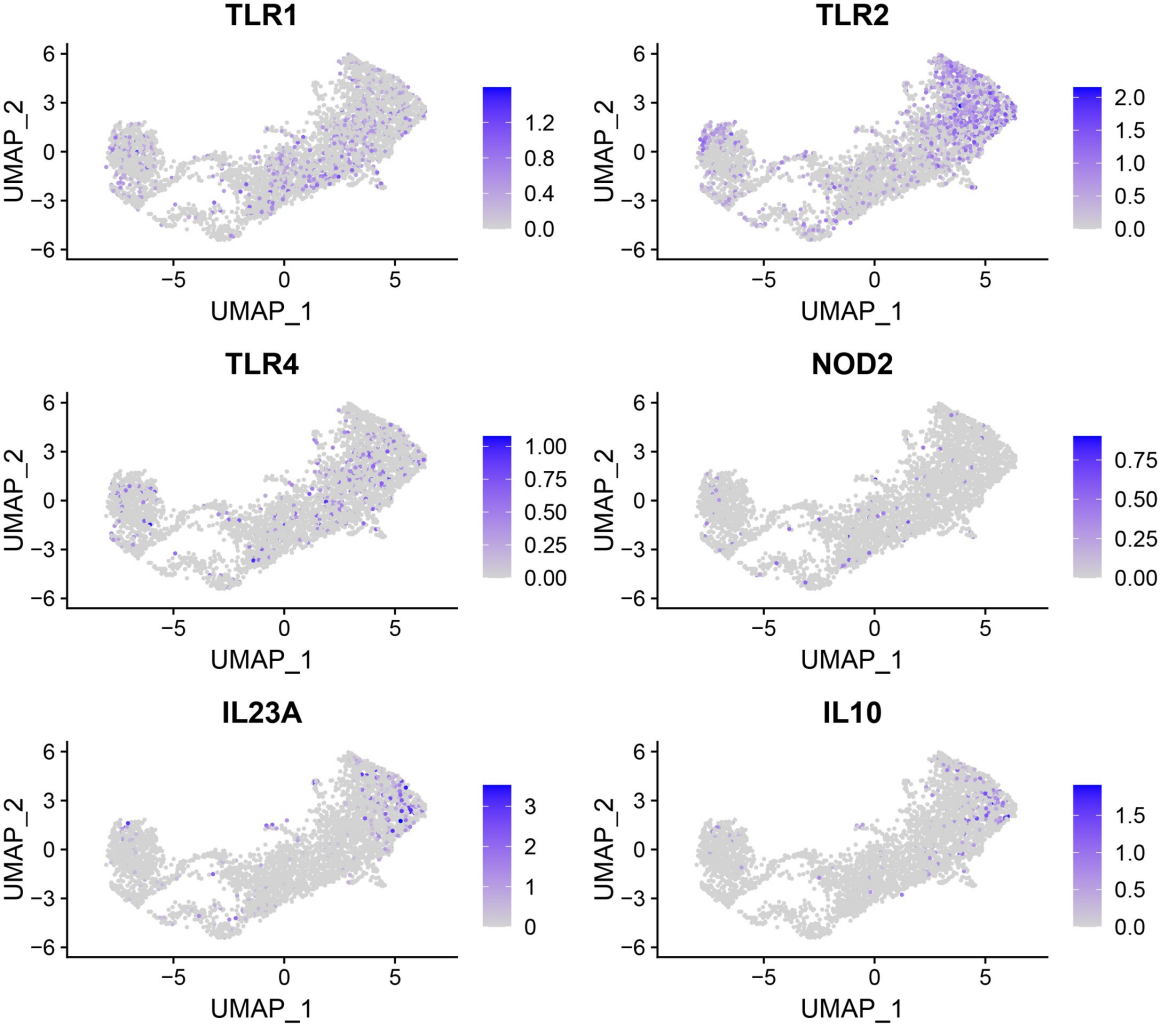
Expression of pattern recognition receptor and cytokine genes in single cells. Cells expressing the corresponding gene were colored according to the expression level (log scale).

In the comparison between cluster 0 and other clusters, the expression of cytokine and chemokine genes such as *IL1B*, *CCL4*, *CCL3*, and *CCL20* was significantly upregulated (Table 1). In the control group of the same cluster, since the proportion of cells expressing the corresponding genes was small, this is considered to be a specific response related to the MAP infection of human macrophages. These genes are activated to defend against infection as part of the innate immune system, and their main role is to recruit neutrophils, T helper cells, and other macrophages to initiate antigen-specific defensive responses. In particular, *CCL3*, *CCL4* and *CCL5* have been reported to be signature markers of Th17 cells in mycobacterial infection [21,22]. Activation of these genes is believed to be due to receptor signaling that induces the expression of the corresponding cytokine. Therefore, the difference in the expression of these genes is expected to be related to the difference in the expression level of the pattern recognition receptor (PRR) by cells in each cluster. Comparison of the expression levels of major PRRs, such as Toll-like receptor (TLR) 1, *TLR2*, *TLR4*, and nucleotide-binding oligomerization domain (NOD) 2, showed that the expression of *TLR2* was increased in cluster 0 cells compared to other clusters (Fig 3).

**Table 1:** Top 20 genes with upregulated expression in cluster 0 compared to other clusters among the infection group.

	p_value	avg_log_2_FC^a^	Pct^b^.cluster_0	Pct^b^.other_clusters	p_val_adjusted
IL1B	5.06E-157	3.002287	0.986	0.708	9.53E-153
CCL4	6.55E-163	2.766326	0.998	0.87	1.23E-158
CCL3	1.84E-155	2.766115	0.994	0.909	3.48E-151
CCL20	3.72E-109	2.378299	0.903	0.546	7.01E-105
MMP9	1.80E-104	2.326808	0.874	0.53	3.39E-100
CXCL3	4.80E-133	2.013032	0.832	0.298	9.04E-129
S100A9	1.17E-55	2.005273	0.623	0.255	2.20E-51
CXCL1	2.28E-100	2.000854	0.801	0.38	4.30E-96
TDO2	6.53E-117	1.995095	0.886	0.433	1.23E-112
SOD2	4.22E-155	1.905559	1	0.897	7.95E-151
IL8	9.87E-117	1.725436	0.994	0.868	1.86E-112
ATP2B1	4.59E-146	1.673085	0.951	0.57	8.66E-142
SPP1	8.21E-78	1.657379	0.972	0.869	1.55E-73
CCL3L3	5.17E-114	1.626342	0.777	0.263	9.74E-110
CD14	1.99E-117	1.50267	0.757	0.242	3.75E-113
TNFAIP6	3.70E-97	1.488727	0.722	0.245	6.97E-93
SAT1	1.17E-106	1.458414	0.99	0.904	2.20E-102
CTSB	6.74E-109	1.393309	0.998	0.952	1.27E-104
CTSL	4.66E-125	1.385936	0.994	0.864	8.78E-121
FTH1	2.67E-179	1.368585	1	1	5.03E-175

^a^Average log2-fold-change.

^b^Proportion of cells expressing that gene.

One example of cluster 0-specific TLR2 expression-related changes was a change in *IL23A* gene expression. *IL23A*, one of the top 10 highly variable features in the infection group, showed a significant increase in expression only in cluster 0 cells. TLR2 is known to activate IL-23p19 together with NOD2 [23–25]; thus, there might be a correlation between *IL23A* expression and TLR2 signaling. Low-level expression of the *IL10* gene, another TLR2-inducible cytokine, was observed mainly in cluster 0 cells with a similar pattern of *IL23A* expression (Fig 3), although it was not identified as a significant gene expression change in cluster 0 due to its low expression level.

## Discussion

In this study, scRNA-seq analysis was performed to observe THP-1 cell differentiation differences in response to PMA treatment and the different responses of these cells to MAP infection. As a result of clustering analysis of THP-1 cells according to their response to PMA treatment, a new cell type (Mreg type) was found in addition to the classification of clusters that appeared to relate to the degree of cell maturation. This result means that different cell types can arise even when the same cell line is treated under the same conditions. Mreg-type cells can be differentiated from CD14^+^ peripheral blood monocytes when cultured with M-CSF and high concentrations of human serum [20]. From the results in this study, DHRS9^+^ cells were differentiated from the THP-1 cell line; thus, PMA treatment might induce the differentiation of Mreg-type cells. However, since the proportion of DHRS9^+^ cells found in this study was very low, optimized treatment conditions are required to use THP-1 cells as a source of Mregs. Mreg macrophage is involved in induction of Treg cells in Mtb infection which impair effector cell function and the host ability to control the infection [26]. In the present study, several DEGs highly expressed in Mreg cells were identified by cluster-specific DEG analysis (S1 File). Although the DEG profile according to MAP infection in Mreg cells was similar to other clusters (S2 File), unique gene expression profile of Mregs might have potential role against MAP infection.

Besides Mregs, there were no other cell clusters that could be defined as new cell types. However, as shown in the comparison among cluster 0 and others, different patterns in gene expression according to MAP infection were observed that originated from the differences in cluster-specific marker genes. For example, *IL23A* expression was specific to cluster 0, and the induction of *IL23A* was due to the expression of TLR2, which is also a cluster 0-specific feature. Therefore, analyzing the gene expression patterns via scRNA-seq classification allows for a more detailed observation of the response to infection by each cell type.

The canonical marker of classically differentiated macrophages is the increased expression of CD11b, CD14, and CD36, as observed in cluster 0 [13,27,28] (S1 Fig). Along with this, TLR2 expression was increased compared to the other clusters, which is another characteristic of classically differentiated macrophages. TLR2 also increases when THP-1 cells are differentiated into macrophages [13]. TLR2 is activated by mannosylated liparabinomannan (ManLAM), the major surface antigen of MAP, and initiates signaling [29]. TLR2 signaling has been thought to be related to the expression of IL-10, a regulatory cytokine associated with MAP infection [30–32]. Moreover, the immune regulatory role of IL-10 has been considered to contribute to the persistence of MAP in the host immune system. Recently, the Th17-like response has been thought to be a major response to MAP infection associated with granuloma formation [33–35]. Early expression of Th17-induced cytokines such as IL-17a is associated with activation of innate T lymphocytes induced by IL-1β, IL-6, and IL-23 produced by phagocytic cells [9]. Although the proportion of cells expressing *IL10* and *IL23A* was low, both genes were found to be expressed following MAP infection.

Because no infection-group-specific cluster was found, the results of the cluster classification in this study mainly indicated the degree of differentiation or maturation of cells in response to PMA treatment as opposed to characterizing the polarization or activation of macrophages in response to MAP infection. There were no observable changes in the expression of canonical marker genes of macrophage polarization to the M1 or M2 state, such as *CD80*, *CD163* and *CD206*. The 3 h infection with MAP may have been too short to observe polarization-related changes, such as M1/M2 macrophage polarization. Nevertheless, classically differentiated cells expressed *CCL3*, *CCL4*, *CC5*, *CXCL1*, *CXCL2*, and *IL1B*, which are known polarizing factors for M1-type macrophages [36]. Therefore, M1 activation along with induction of Th1/Th17 activation might be specific responses of human macrophages against MAP infection at the initial stage. In further studies, a cluster-based analysis involving the polarization of THP-1 cells exposed to MAP for a longer time will test this hypothesis.

In this study, the differentiation characteristics of THP-1 cells in response to PMA treatment were analyzed using a novel transcriptomic ‘snap-shot’ tool, scRNA-seq. The analysis identified a cell type that has never been reported to differentiate from THP-1. Furthermore, genes significantly highly expressed in specific cell clusters, such as CD52, RAMP1, and PTGS1, were identified as cluster specific biomarkers. Although the functional implications of expression of those genes in THP-1 in response to PMA treatment are not clear, our results including trajectory analysis suggesting that those genes have potential role in THP-1 differentiation. Infection of PMA-treated THP-1 cells with MAP showed that the CD14^+^ cluster (classically differentiated macrophages) was most responsive to MAP, and this response was suggested to be associated with significantly higher TLR2 expression in that cluster. In conclusion, our results provide a basis for understanding the interactions between MAP and the human immune system.

## Supporting information

S1 Fig Gene expression level of canonical markers indicating macrophage differentiation. (TIF)

S1 File Differential gene expression profiles in the cluster compared to the other clusters. (XLSX)

S2 File Differential gene expression profiles in the infected group compared to the control group in each cluster. (XLSX)
