# Supplementary figures and images for: Revealing immune responses in the Mycobacterium avium subsp. paratuberculosis-infected THP-1 cells using single cell RNA-sequencing
Source: PLoS One. 2021 Jul 2;16(7):e0254194. doi: 10.1371/journal.pone.0254194 (PMC8253428; doi:10.1371/journal.pone.0254194)

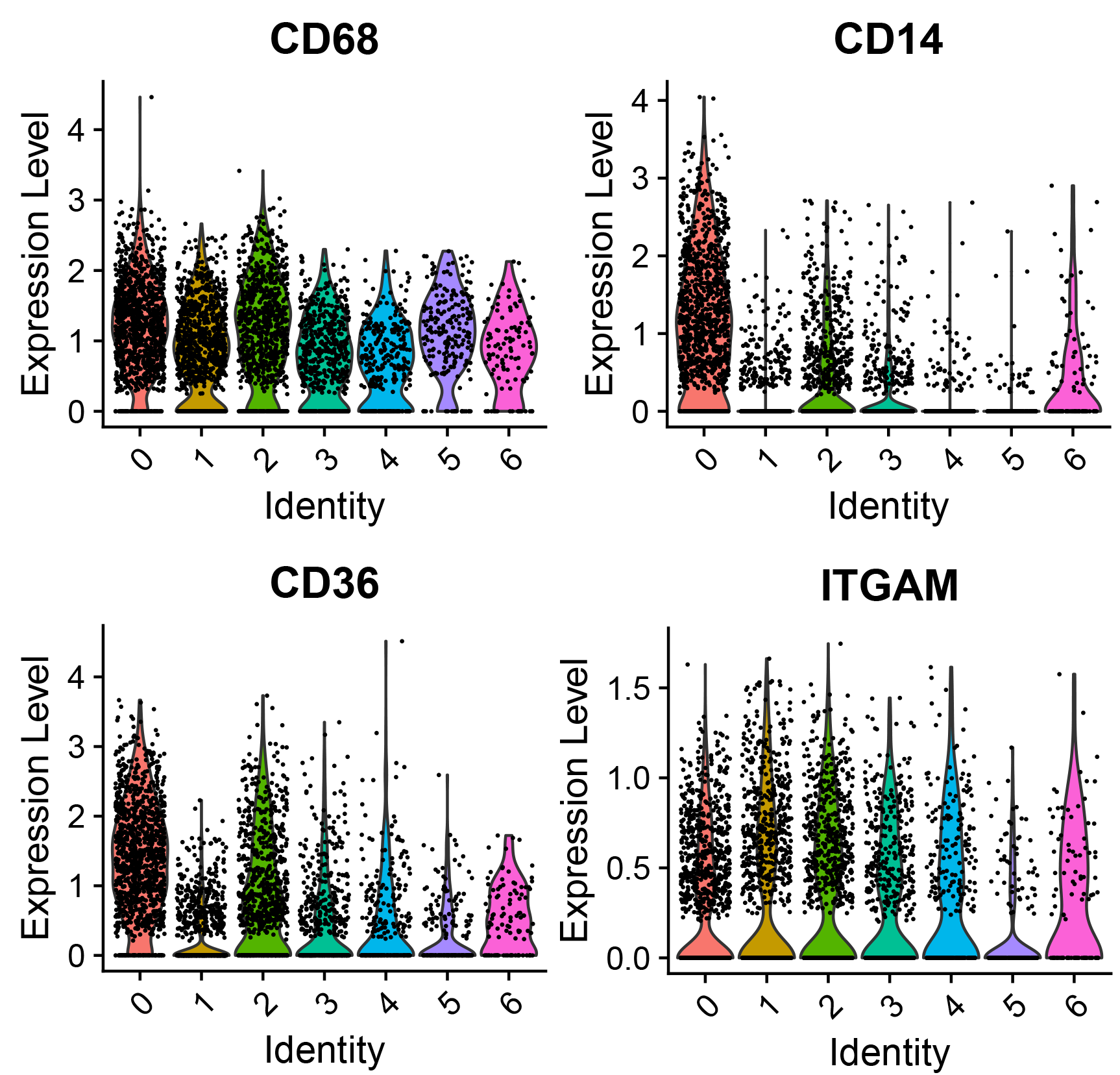

Supplement: S1 Fig — (TIF) [file pone.0254194.s001.tif]
